# Transcriptomic SNP discovery for custom genotyping arrays: impacts of sequence data, SNP calling method and genotyping technology on the probability of validation success

**DOI:** 10.1186/s13104-016-2209-x

**Published:** 2016-08-26

**Authors:** Emily Humble, Michael A. S. Thorne, Jaume Forcada, Joseph I. Hoffman

**Affiliations:** 1 Department of Animal Behaviour, University of Bielefeld, Postfach 100131, 33501 Bielefeld, Germany; 2 British Antarctic Survey, High Cross, Madingley Road, Cambridge, CB3 OET UK

**Keywords:** Transcriptome, Roche 454 sequencing, Illumina HiSeq sequencing, Single nucleotide polymorphism, Validation success, Marine mammal, Antarctic fur seal, *Arctocephalus gazella*

## Abstract

**Background:**

Single nucleotide polymorphism (SNP) discovery is an important goal of many studies. However, the number of ‘putative’ SNPs discovered from a sequence resource may not provide a reliable indication of the number that will successfully validate with a given genotyping technology. For this it may be necessary to account for factors such as the method used for SNP discovery and the type of sequence data from which it originates, suitability of the SNP flanking sequences for probe design, and genomic context. To explore the relative importance of these and other factors, we used Illumina sequencing to augment an existing Roche 454 transcriptome assembly for the Antarctic fur seal (*Arctocephalus gazella*). We then mapped the raw Illumina reads to the new hybrid transcriptome using BWA and BOWTIE2 before calling SNPs with GATK. The resulting markers were pooled with two existing sets of SNPs called from the original 454 assembly using NEWBLER and SWAP454. Finally, we explored the extent to which SNPs discovered using these four methods overlapped and predicted the corresponding validation outcomes for both Illumina Infinium iSelect HD and Affymetrix Axiom arrays.

**Results:**

Collating markers across all discovery methods resulted in a global list of 34,718 SNPs. However, concordance between the methods was surprisingly poor, with only 51.0 % of SNPs being discovered by more than one method and 13.5 % being called from both the 454 and Illumina datasets. Using a predictive modeling approach, we could also show that SNPs called from the Illumina data were on average more likely to successfully validate, as were SNPs called by more than one method. Above and beyond this pattern, predicted validation outcomes were also consistently better for Affymetrix Axiom arrays.

**Conclusions:**

Our results suggest that focusing on SNPs called by more than one method could potentially improve validation outcomes. They also highlight possible differences between alternative genotyping technologies that could be explored in future studies of non-model organisms.

**Electronic supplementary material:**

The online version of this article (doi:10.1186/s13104-016-2209-x) contains supplementary material, which is available to authorized users.

## Background

High throughput sequencing and cost efficient genotyping technologies are revolutionising the study of wild organisms [1]. For example, many thousands of single nucleotide polymorphisms (SNPs) can now be genotyped in virtually any organism [2, 3]. Although individually less informative than multi-allelic markers, SNPs are appealing because they can be genotyped rapidly, in large numbers and with minimal error [4, 5]. Consequently, SNP datasets are being generated for an increasing number of wild animal populations, allowing researchers to address a variety of outstanding questions in evolutionary biology, conservation genetics and wildlife management [6–8].

In non-model species, SNPs are often mined from transcriptome assemblies, as these are smaller and simpler to generate than genomes. Nevertheless, there are a variety of alternative methods available for read mapping and variant discovery and it is not always straightforward to know which of these to use. Relatively few systematic comparisons of the available programs have been carried out and most have mainly been based on genomic data from humans [9, 10]. These studies suggest that in some cases the concordance between different methods can be poor [11, 12], yet it is still the norm to call SNPs with a single method [13–15]. By drawing upon vast numbers of previously known SNPs, human studies have also evaluated the relative success of different methods at discovering known variants [16]. However, less attention has been paid to non-model organisms, partly because for many species, SNPs are being discovered for the first time.

SNP discovery can facilitate multiple genotyping approaches. Genotyping by sequencing approaches such as RAD, ddRAD and 2bRAD [17–19] allow simultaneous SNP discovery and genotyping. These approaches enable studies to scale up to much larger sample sizes of individuals and loci than what was possible with traditional markers such as microsatellites. However, large amounts of high quality DNA are required, library preparation can be costly and labour intensive, and downstream analyses are not straightforward [20]. High density SNP arrays, or ‘SNP chips’, have thus become increasingly popular for large-scale studies, as they are relatively cheap per sample, technically more straightforward, allow selected SNPs to be consistently genotyped across the majority of individuals, and enable candidate genes to be targeted [21, 22]. However, careful selection of SNPs is necessary as not all ‘putative’ SNPs will be suitable for genotyping. For example, SNPs must have sufficient flanking sequence that is compatible with a given genotyping technology. The two most widely used array platforms, Illumina Infinium iSelect HD [23] and Affymetrix Axiom [24], implement distinctive hybridization technologies and require probes of different lengths. Moreover, recent studies suggest that the genomic context of a SNP can have a significant impact on validation success [25, 26], defined as the propensity of a given SNP to be polymorphic and reliably scored in a sample of individuals. For example, transcripts representing paralogous genes can result in SNP probe sequences that map many times to a genome, whilst probe sequences inadvertently spanning intron–exon boundaries will result in failure of the probe to bind to the genomic DNA [25, 27–29]. By mapping SNP flanking sequences to reference genomes, both of these issues have been shown to have a significant impact on validation success [25, 26, 30–32].

An opportunity to quantify the extent of overlap between different SNP discovery methods and to explore the consequences for validation success is provided by a study of Antarctic fur seals (*Arctocephalus gazella*). A transcriptome assembly based on Roche 454 sequencing is already available for this species, from which two SNP datasets were generated using NEWBLER and SWAP454 respectively [33, 34]. Here, we supplement this transcriptome with short read Illumina sequencing, allowing a comparison of SNP discovery methods tailored to different types of sequence data. We also recently developed a predictive modeling framework to determine the likelihood of validation success by accounting for a variety of variables, from compatibility of the probe sequences with a given assay chemistry, through in silico features such as depth of coverage and minor allele frequency (MAF), to aspects of the genomic context [26]. This framework provides a basis by which we can evaluate the likely validation outcomes of the SNPs discovered by different methods.

In this study, we first generated a ‘hybrid’ fur seal transcriptome from the 454 and Illumina data. We then mapped the Illumina reads to the hybrid transcriptome using BWA and BOWTIE2 before calling SNPs from each alignment with GATK. The two sets of resulting SNPs were then compared with the two sets of SNPs previously mined from the 454 transcriptome using NEWBLER and SWAP454 respectively. This allowed a direct comparison of a total of four methods for calling SNPs from two types of sequence data. Finally, we used predictive modeling to assess the suitability of the resulting SNPs for both an Affymetrix Axiom and an Illumina Infinium iSelect HD array. We hypothesized that SNPs with a high probability of validation success would be enriched for those called by more than one method. Due to the higher depth of coverage provided by Illumina relative to 454 sequencing, we also expected SNPs called from the former to have higher validation success probabilities. We provide an annotated workflow within the R programming language [35] for implementing the SNP filtering and assessment steps presented here (Additional file 1).

## Results

### Sequencing, assembly and annotation

**Fig. 1:**
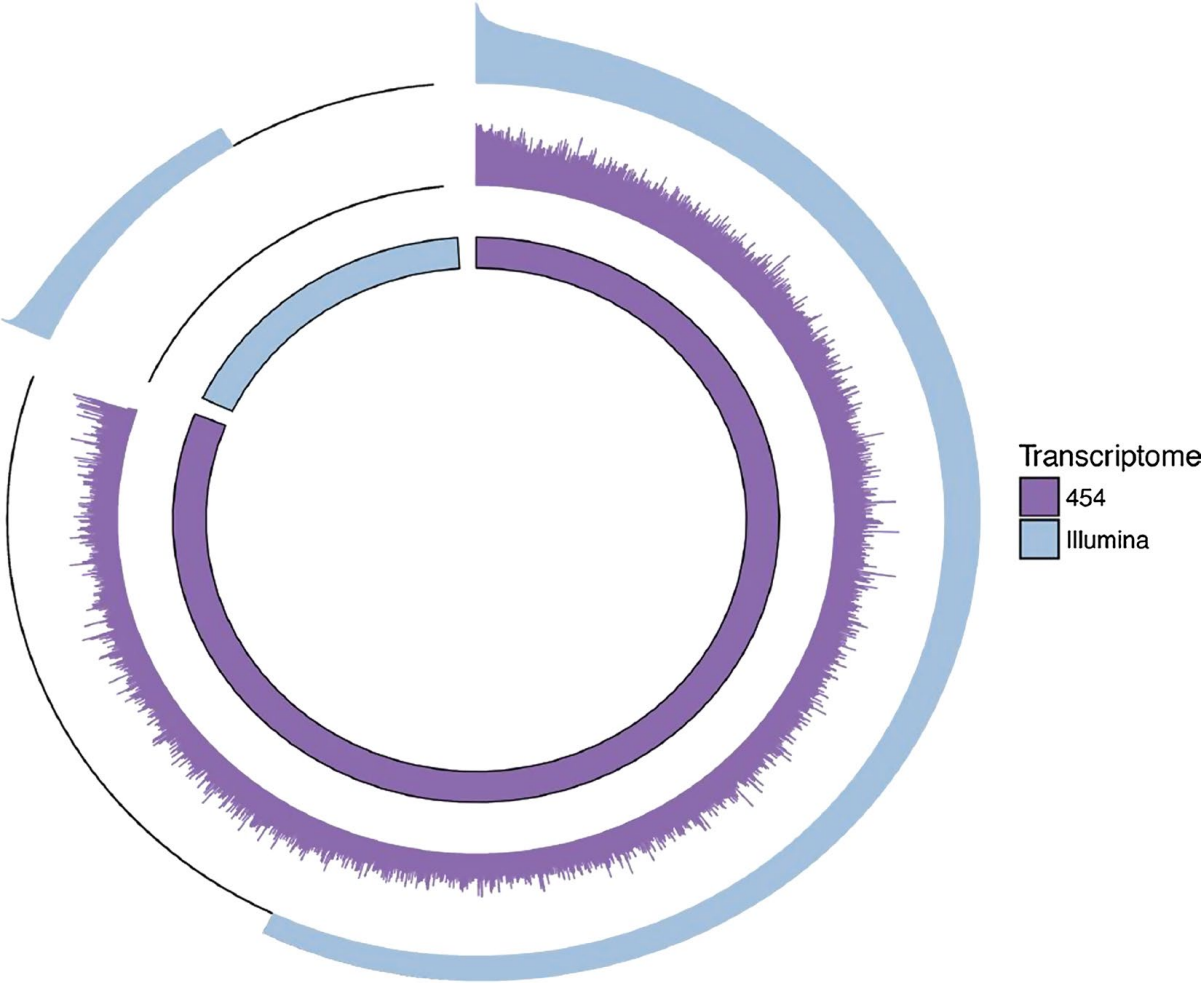
Circular plot showing the hybrid transcriptome assembly. The *inner track* represents the breakdown of the transcriptome into 454 (*purple*) and Illumina (*blue*) components. The *middle* and *outer tracks* show the depth of coverage of the 454 and Illumina reads plotted on a log scale. Transcripts are sorted in order of average Illumina coverage. As we required at least ten fold Illumina coverage of a given nucleotide to call a SNP, Illumina coverage of transcripts with less than tenfold average coverage has been truncated zero

To improve upon the existing 454 transcriptome, which comprises 23,096 contigs of mean length 971 bp, we conducted an additional round of Illumina sequencing (see ‘Methods’ section for details). This generated a total of 17,894,042 101 bp paired-end reads (submitted to the sequence read archive, http://www.ncbi.nlm.nih.gov/sra; Study Accession SRP071273), which were assembled de novo to generate 26,266 contigs of mean length 904 bp. Blasting these contigs to the 454 backbone, we found that 15,520 (59.0 %) successfully mapped at an e-value threshold of 1e^−10^. After annotating the unmapped contigs, around 50 % were removed, either due to a lack of homology to known sequences or because the top BLAST hit revealed similarity to known bacterial or viral sequences. Most of the remaining 5452 annotated contigs showed sequence similarity to the Weddell seal (*Leptonychotes weddellii*) and the walrus (*Odobenus rosmarus*) and were thus concatenated to the original 454 transcriptome. This yielded a ‘hybrid transcriptome’ comprising a total of 28,548 contigs (Fig. 1, http://www.goo.gl/vj8VjD). To investigate homology to the dog (*Canis familiaris*), we mapped these contigs to the most recent and complete build of the dog transcriptome. 23,587 (82.6 %) of the seal contigs mapped to 35,724 (64.7 %) of the dog transcripts, suggesting that a reasonably large proportion of the fur seal transcriptome has been captured.

### Overlap in SNP discovery

**Fig. 2:**
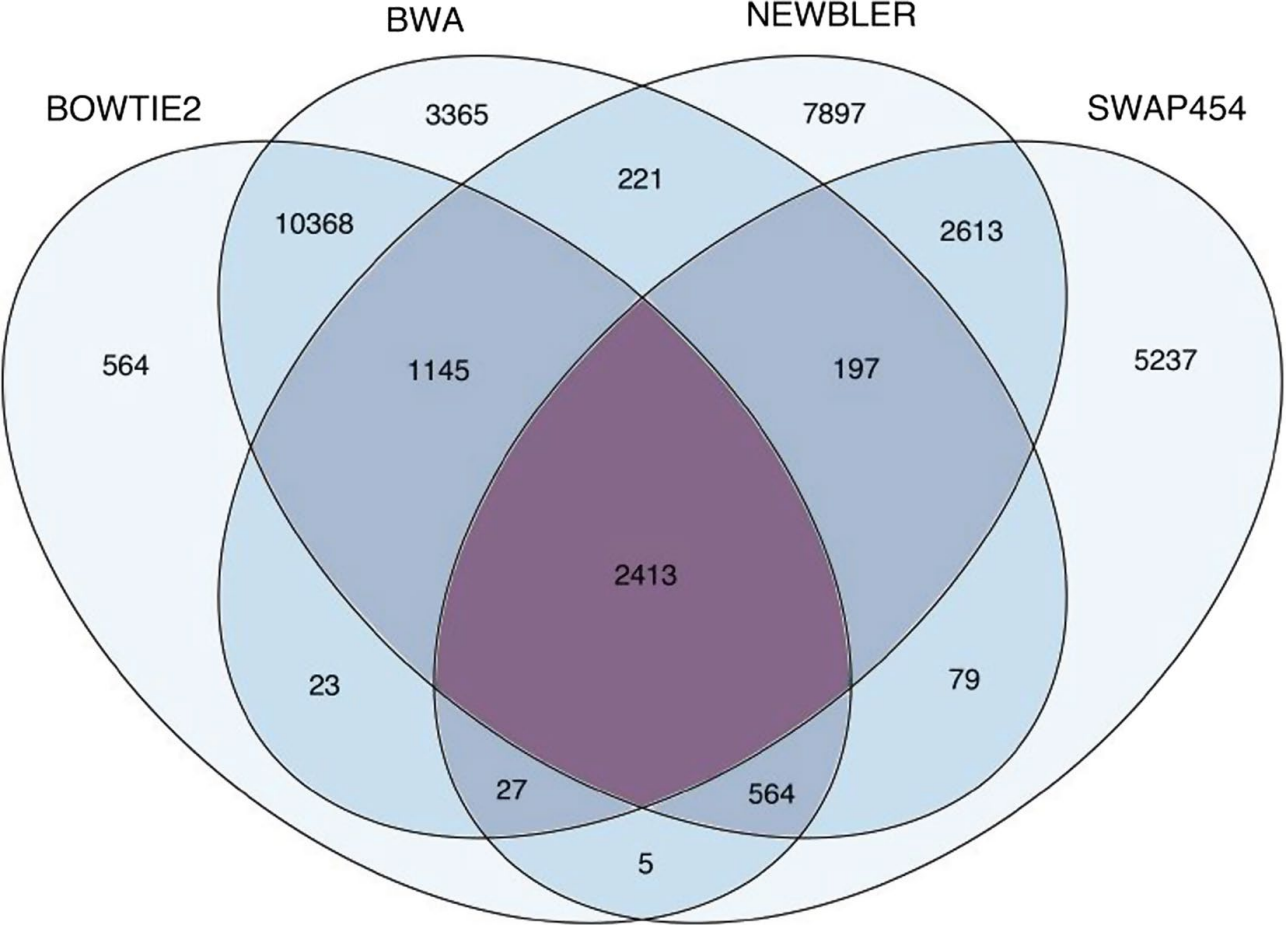
Venn diagram showing the extent of overlap among SNPs called using four different methods (see ‘Methods’ section for details)

The 454 transcriptome was previously mined for SNPs using NEWBLER and SWAP454, which identified 14,538 and 11,155 SNPs respectively [34]. To call SNPs from the Illumina data, we mapped the raw Illumina reads to the hybrid transcriptome using BWA and BOWTIE2 and parsed the resulting alignment files to GATK as described in the ‘Methods’ section. This resulted in a total of 18,353 SNPs from the BWA alignment and 15,109 from the BOWTIE2 alignment, of which 14,490 SNPs were called by both methods. Pooling SNPs across all four methods resulted in a dataset of 34,718 unique markers. To explore the extent of overlap between the SNP calling methods described above we generated a Venn diagram (Fig. 2). This shows that 49.1 % of the total 34,718 SNPs were called by a single method, 38.3 % were called by two methods, 4.6 % by three and 7.0 % by all four. Most of the SNPs identified by a single method (76.9 %) were called from the 454 transcriptome using NEWBLER or SWAP454. The overlap between SNPs discovered from the 454 and Illumina data was 13.5 %.

### SNP parameter space

**Fig. 3:**
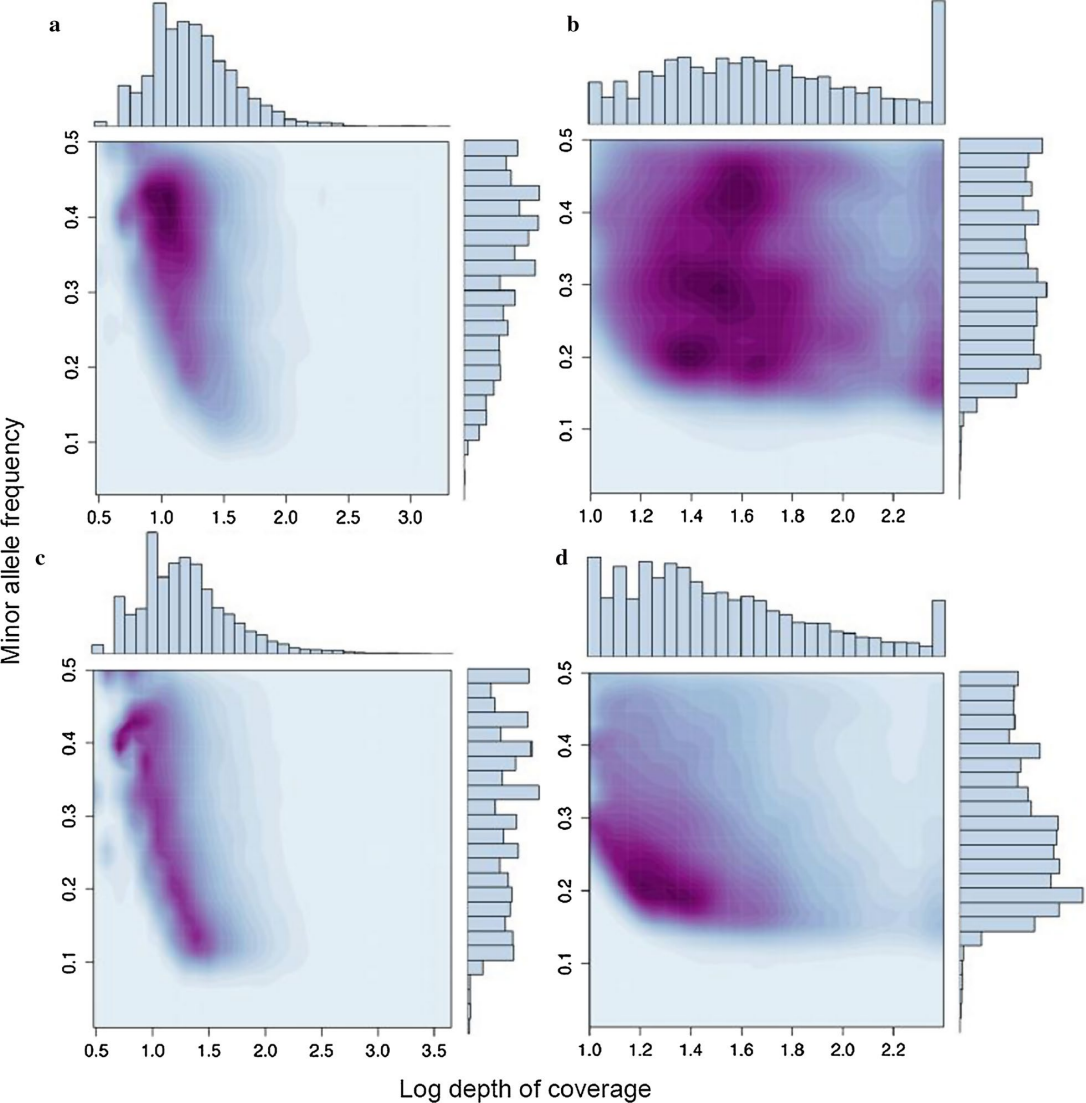
Variation in SNP minor allele frequency (MAF) and depth of sequence coverage. The *upper panels* correspond to 4679 SNPs that were called from both the 454 and Illumina datasets, with panel **a** showing the 454 parameter space and **b** showing the corresponding Illumina parameter space. The *lower panels* correspond to the total number of SNPs called from the 454 and Illumina data (20,426 and 18,971 respectively), with panel **c** showing the 454 parameter space and **d** showing the corresponding Illumina parameter space

The increased depth of coverage provided by Illumina sequencing should allow in silico minor allele frequency (MAF) to be estimated more accurately than for the 454 data. We therefore selected the subset of 4679 SNPs that were called from both the 454 and Illumina datasets and compared their respective parameter spaces. Two obvious differences emerge between the two datasets (Fig. 3). First, average log depth of coverage of the SNPs increases substantially, from around 1.2 (corresponding to 16× coverage) for the 454 data to 1.7 (corresponding to 50× coverage) for the Illumina data. Second, we find a marked difference in the respective MAF distributions, which are concentrated around 0.4 for the 454 data (Fig. 3a) but which are more evenly spread between around 0.2 and 0.5 for the Illumina data (Fig. 3b).

We also used the same approach to compare all of the SNPs called from the 454 data with all of the SNPs called from the Illumina data. Again we found marked differences between the two datasets (Fig. 3). For the 454 data, a clear relationship emerged between MAF and depth of coverage, SNPs with high MAF mainly being called at a relatively low depth of coverage, whereas SNPs with low MAF were mainly called at a relatively high depth of coverage (Fig. 3c). For the Illumina data, SNPs were predominantly called at a relatively low depth of coverage (Fig. 3d), which is probably a more accurate approximation of the underlying MAF distribution (see ‘Discussion’ section).

### SNP filtering and predicted assay success

**Fig. 4:**
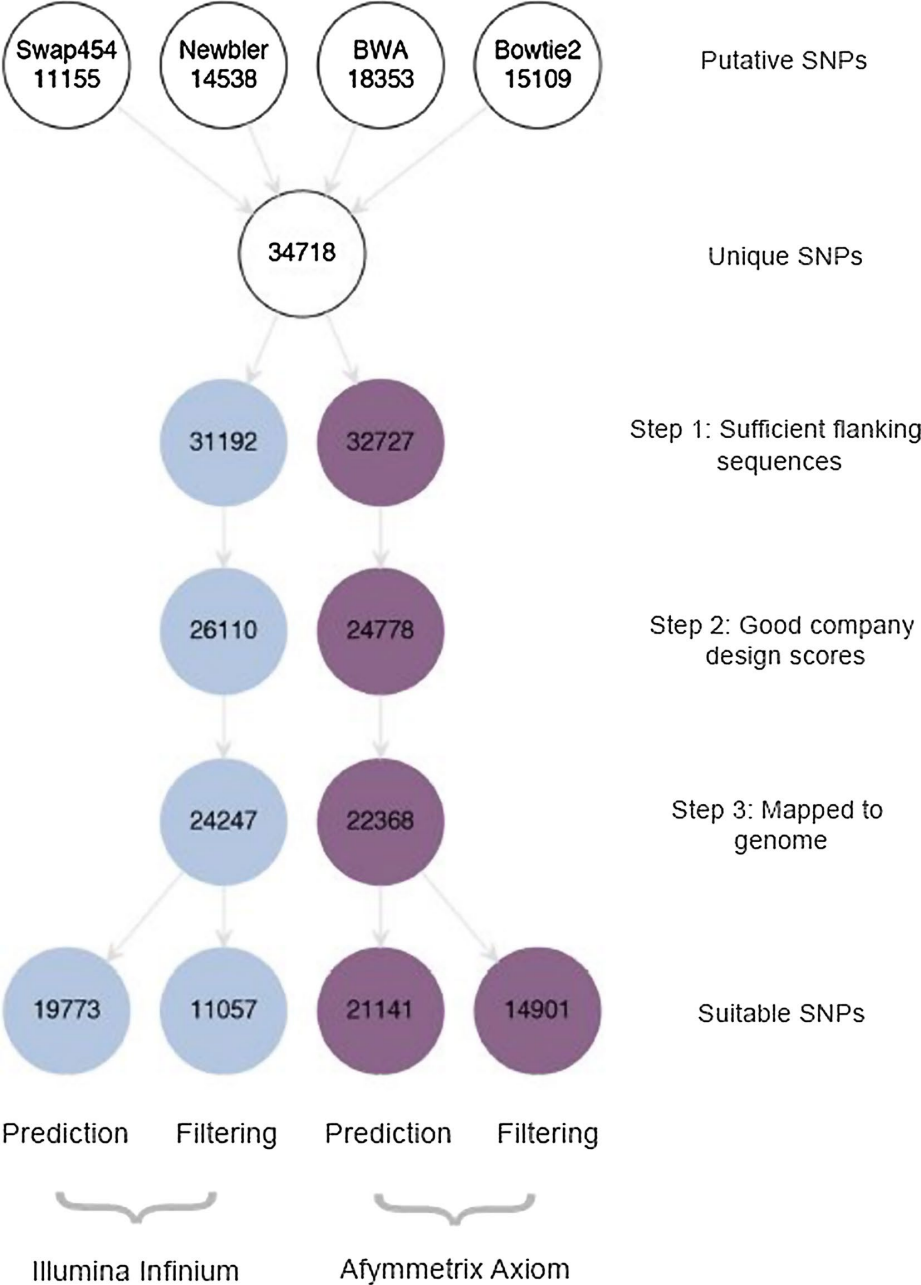
Flow diagram showing the number of SNPs remaining after each step of the SNP detection pipeline for both an Illumina Infinium iSelect HD array (*blue circles*) and an Affymetrix Axiom array (*purple circles*)

Although most studies present the total number of putative SNPs identified from transcriptome assemblies, when developing a custom SNP array it is important to consider the likelihood of each SNP successfully validating with a given genotyping technology. We therefore tested the total set of 34,718 SNPs for compatibility with both Illumina Infinium iSelect HD and Affymetrix Axiom high density SNP arrays (Fig. 4). In order to do this, we extracted the flanking sequences required for Infinium iSelect (121 bp) and Affymetrix Axiom (71 bp) probe design from the fur seal transcriptome. Complete 121 bp flanking sequences could be extracted for 31,192 of the SNPs (89.8 %) while the equivalent proportion was slightly higher for the 71 bp flanking sequences (*n* = 32,727, 94.3 %, Step 1 in Fig. 4). The Illumina and Affymetrix flanking sequences were then evaluated using Illumina’s Assay Design Tool (ADT) and Affymetrix’s SNP evaluation pipeline respectively. 26,110 SNPs (86.6 %) were assigned ADT scores of ≥0.8 and 24,778 (78.4 %) were classified as either ‘recommended’ or ‘neutral’ by Affymetrix (Step 2 in Fig. 4).

Following this, we sought to remove SNPs with an undesirable genomic context by mapping their flanking sequences to the draft fur seal genome (Step 3 in Fig. 4). Blasting the Infinium and Affymetrix SNP sequences with an e-value threshold of 1e^−12^ recovered 24,247 and 22,368 hits respectively. For these SNPs, we evaluated the probability of successful validation using a predictive model incorporating MAF, depth of coverage, ADT/p-convert score plus values of the predictor variables generated from the genome BLAST (see ‘Methods’ section for details). Based on the 121 bp Infinium sequences, 19,773 (81.5 %) SNPs were predicted to successfully validate with a probability threshold of 0.7. Both the number (21,141) and proportion (94.5 %) of equivalent Affymetrix sequences were higher. Simply filtering the flanking sequences for those that mapped completely and uniquely to the reference genome resulted in fewer SNPs being retained (11,057 Illumina flanking sequences and 14,901 Affymetrix flanking sequences, Fig. 4).

We next asked whether the probability of successful validation varied according to SNP calling method. Of the SNPs called from the 454 data using NEWBLER and SWAP454, only 46.8 and 57.0 % respectively were predicted to successfully validate when using an Illumina assay (Table 1). By contrast, 75.7 % of SNPs called by GATK from the BOWTIE2 alignment and 72.1 % from the BWA alignment were predicted to successfully validate. A similar pattern was obtained when considering SNPs that map completely and uniquely to the reference genome, as well as for predictive models based on the Affymetrix flanking sequences (Table 1).

**Table 1:** Proportion of SNPs from each discovery method predicted to successfully validate on both an Illumina Infinium and an Affymetrix Axiom array using predictive modeling and simple filtering approaches

Discovery method	Predicted validation success (%)			
	Infinium		Axiom	
	Predictive	Filtering	Predictive	Filtering
BOWTIE2	75.7	45.6	83.3	61.7
BWA	72.1	39.7	78.5	54.6
NEWBLER	46.8	27.3	48.9	35.5
SWAP454	57.0	34.6	61.8	45.9

Finally, we tested whether the probability of successful validation varied with the number of methods by which a given SNP was called. Table 2 shows that, when using an Illumina assay, regardless of whether a predictive modeling or simple filtering approach is taken, predicted validation success rates are around one-third to two times higher for SNPs called by two or more methods, with those called by two methods yielding the greatest predicted validation success. The same pattern is found for the Affymetrix flanking sequences, although the predicted outcomes are somewhat less dependent on the number of methods by which a SNP is called.

**Table 2:** Proportion of those SNPs shared by one, two, three and four calling methods predicted to successfully validate on both an Illumina Infinium and an Affymetrix Axiom array using using predictive modeling and simple filtering approaches

Share	Predicted validation success (%)			
	Infinium		Axiom	
	Predictive	Filtering	Predictive	Filtering
One	66.8	30.7	91.7	57.0
Two	92.9	57.2	96.7	72.6
Three	89.3	52.5	93.2	68.0
Four	89.9	54.0	93.3	68.2

## Discussion

We used Illumina sequencing to augment an existing fur seal transcriptome assembly generated from 454 sequence data. We then attempted to maximise successful SNP discovery both by exploring the overlap between SNPs called using four different methods and by evaluating predicted validation outcomes. We found that SNPs called from the Illumina data were on average more likely to successfully validate, as were SNPs called by more than one method. Predicted validation outcomes were also found to be slightly better for Affymetrix Axiom than Illumina Infinium iSelect HD arrays.

### The hybrid transcriptome assembly

We de novo assembled the Illumina HiSeq data into contigs and then mapped these to the 454 backbone. Over 5000 additional contigs were generated that revealed homology to walrus and Weddell seal sequences, suggesting that the hybrid assembly is more complete than the 454 assembly (Fig. 1). To explore this further, we mapped the fur seal contigs to the most recent and complete build of the dog transcriptome. We found that 82.6 % of the contigs mapped to 64.7 % of the dog transcripts. This is in contrast to what was previously reported for the 454 transcriptome, where 62.5 % of seal contigs mapped to 77 % of dog transcripts [34]. Therefore, whilst a greater proportion of the transcriptome is mapping, a slightly smaller fraction of the dog transcriptome is represented. This is probably because the mapping was performed against a more recent and complete build of the dog transcriptome.

### SNP discovery

The greater depth of coverage and improved representation of fur seal transcripts provided by Illumina sequencing provides the opportunity both to increase the total pool of SNPs discovered and to cross-check SNPs called from the 454 and Illumina data. In this study, we compared four different methods for mining SNPs from two different types of sequence data. Specifically, 454 data were mined for SNPs using NEWBLER and SWAP454, whilst GATK was used to mine SNPs from both a BWA and a BOWTIE2 Illumina read alignment. We found poor concordance between the SNPs discovered by all four methods, with only 51.0 % of SNPs being discovered by two or more methods. This is consistent with previous studies, mostly based on genomic data from humans, which have also found relatively little overlap between SNPs called by different tools [12, 16] although few of these studies attempted to explore validation outcomes as we have done here.

There are several potential explanations for the limited overlap between SNPs called from the 454 and Illumina datasets. First, the hybrid transcriptome contains around 5000 contigs that are only represented by Illumina sequences and from which any called SNPs will therefore be unique. However, these only account for 5.7 % of the total number of Illumina-specific SNPs, suggesting that the majority are located within contigs that are also represented by 454 data. Thus, it seems likely that Illumina sequencing allowed many more SNPs to be called from the same contigs by virtue of the increased depth of coverage provided. This is supported by two lines of evidence. First, the median depth of coverage of SNPs called from the Illumina data was 28, whereas the equivalent was only 16 for the 454 data. Second, we observed a shift towards SNPs with relatively low minor allele frequencies being called from the Illumina data, suggesting that greater depth of coverage facilitates the discovery of such polymorphisms.

A second reason for the limited overlap could be that the 454 transcriptome includes both skin and necropsy samples whereas for the current round of Illumina sequencing we were only able to use remaining cDNA from the skin samples. Thus, the 454-specific SNPs were called from both the skin and necropsy parts of the transcriptome, whereas the Illumina-specific SNPs were only called from the skin part. Indeed, for both BWA and BOWTIE2 alignments, not all of the 454 transcriptome was mapped to by the Illumina reads (Fig. 1); around 40 % was left with insufficient Illumina sequence coverage for SNP calling, presumably because it represented necropsy-specific transcripts. Another possibility is that not all of the SNPs called from the 454 data may be genuine. In support of this, only 25.6 % of the 454-specific SNPs were called by both NEWBLER and SWAP454, suggesting that the two programs differ considerably in their outputs even for the same sequence resource.

Regardless of the differences between SNPs called from the 454 and Illumina data, it is noteworthy that we also found some degree of overlap. Almost 5000 SNPs in total were called from what are essentially independent sequence datasets. For this reason, we consider these SNPs more likely to be genuine, consistent with the finding that SNPs called by more than one method are more likely to be suitable for use in a high density SNP array (see below). Direct comparison of SNPs called from the 454 and Illumina data also revealed marked differences in their MAF distributions, the former being dominated by SNPs with a MAF of around 0.4 while the latter show a more even MAF distribution. While we cannot yet say which of these is the most accurate portrayal of the true underlying distribution, we suspect that the Illumina data are closer to the mark because, at least in theory, greater depth of coverage should allow in silico allele frequency distributions to be estimated more accurately. This finding could thus explain why studies often find no association between in silico and realised allele frequencies [36–38].

### Exploring validation success

SNP discovery is an important goal of many studies and features prominently in many publications describing transcriptomes [39–41]. However, the resulting SNPs may not provide a reliable indication of the number that are likely to successfully validate with a given genotyping technology. For this it is necessary to account for variables such as (i) the proportion of SNPs for which complete flanking sequences can be extracted; (ii) compatibility of the SNP flanking sequences with the chosen assay chemistry; (iii) variation in the likelihood of a SNP being genuine with MAF and depth of coverage; and (iv) aspects of the genomic context including sequence uniqueness and proximity to intron–exon boundaries. We therefore incorporated the above factors into the predictive framework of Humble et al. [26] to evaluate the probability of each SNP successfully validating on both Illumina Infinium iSelect HD and Affymetrix Axiom genotyping arrays. A number of patterns emerged. First, the proportion of SNPs for which complete flanking sequences could be extracted was lower for Illumina than Affymetrix (86.8 versus 91.0 % respectively) reflecting Illumina’s requirement for substantially longer flanking sequences (121 versus 71 bp respectively) for probe design. Second, a larger proportion of SNPs was deemed suitable for assay design based on Illumina ADT scores than Affymetrix p-convert scores (86.6 versus 78.4 % respectively). This pattern is reflected in the proportion of SNPs predicted to successfully validate with each technology, which was over ten percent higher for Affymetrix (94.5 %) than Illumina (81.5 %). Although Illumina require longer flanking sequences for assay design, the probes themselves are only 60 bp long (plus a one base terminal SNP site). Therefore, the difference in predicted validation rates seems unlikely to be related to probe length. Instead, it could be possible that Affymetrix’s evaluation pipeline is more stringent, potentially in this case because it utilized the fur seal genome to determine strand specificity. Regardless of the exact reasons, our findings suggest that under certain circumstances Affymetrix Axiom genotyping arrays might be preferable in some respects to Illumina Infinium iSelect HD arrays, particularly when genotyping non-model organisms with SNPs that have not been experimentally validated in advance.

We also tested whether the probability of successful validation varied according to the method by which a given SNP was called. Above and beyond the pattern described above, we found that SNPs called only from the 454 data (using either NEWBLER or SWAP454) were less likely on average to successfully validate than SNPs called only from the Illumina data (using BOWTIE2 or BWA in combination with GATK). This suggests that a larger proportion of SNPs called from the 454 data may be spurious, in line with the lower depth of coverage of the 454 data, the fact that only around a quarter of these SNPs were called by both NEWBLER and SWAP454, and the limited overlap between SNPs called from the 454 and Illumina data. This finding would also be consistent with our previous work on fur seals in which we experimentally validated a panel of putative SNPs derived from the 454 transcriptome using Illumina’s GoldenGate assay [37]. This study found a positive relationship between in silico MAF and validation success, which suggests that some of the assays may have been designed from paralogous loci.

Finally, we found that the probability of successful validation was greater for SNPs detected using more than one method than for SNPs flagged by a single method. The highest overall validation success rate was obtained for SNPs called by two methods while a marginal reduction was found for SNPs called by three or four methods. To explore this further, we calculated the proportion of the total number of SNPs called by each of the four methods separately for SNPs called by one, two, three or four methods respectively. We found that the peak in validation success corresponding to SNPs called by two methods can be explained by a greater proportion of those SNPs having been called by GATK after mapping with either BOWTIE2 or BWA (Additional file 1: Figure S1). By contrast, SNPs called by three or four methods were more likely to have been called by NEWBLER or SWAP454. As previously discussed, the latter may be of lower average quality and therefore appear to contribute towards a slight deterioration in predicted validation success rates for SNPs called by three and four methods.

Despite the above, a general tendency for SNPs called by more than one method to be more likely to successfully validate makes good sense because the more methods that are used to call a given SNP, the more robust that SNP should be to the peculiarities of any single computer program. Thus, we would advocate the use of more than one SNP calling method as a means of identifying the most robust SNPs, particularly when resources are limited and a high rate of validation success is an important outcome. Overall, our results also highlight how Illumina sequencing is preferable for SNP discovery given the substantially greater depth of coverage that it provides.

## Conclusions

We used Illumina sequencing to improve upon an existing fur seal transcriptome assembly. We then attempted to maximise successful SNP discovery both by exploring the overlap between SNPs called using four different methods and by evaluating predicted validation outcomes. We found that SNPs called from the Illumina data had higher likelihoods of successful validation, as did SNPs called by more than one method. Predicted validation outcomes were also found to be consistently better for Affymetrix Axiom than Illumina Infinium iSelect HD arrays. One possible means of exploring the relative merits of these two genotyping technologies would be to genotype a set of individuals and SNPs using both technologies.

## Methods

### Initial transcriptome

This study partly builds upon a previously published fur seal transcriptome assembly. This was constructed using 454 sequence reads generated from two different cDNA libraries, one comprising skin samples from 12 individuals [33] and the other comprising necropsy samples from nine individuals [34]. Assembly of these data using NEWBLER generated a total of 23,096 contigs [34], which we refer to as the ‘454 transcriptome’.

### Library preparation and Illumina sequencing

Using RNA from the same 12 individuals used for the skin transcriptome, we generated cDNA libraries using Illumina’s TruSeq^®^ Stranded protocol. Briefly, poly-A containing mRNA molecules were purified from the pooled total RNA using oligo-dT beads. The mRNA was subsequently fragmented and reverse transcribed into cDNA with strand specificity. Adaptors and a single ‘A’ base were attached to each fragment before purificiation and PCR enrichment in order to generate the final cDNA library. This was sequenced on one lane of an Illumina HiSeq 2000.

### Sequence assembly

Raw sequencing reads with a Phred quality score of less than 20 were removed and primer and adaptor sequences were trimmed prior to assembly. Cleaned reads were assembled together using SOAPdenovo. After running a range of kmer sizes to determine the optimal k value for contig length and number, the kmer run of 23 was chosen. Only transcripts of length greater than 500 bp were retained in the final assembly.

### Mapping and sequence annotation

All newly generated Illumina contigs were mapped to the previously assembled 454 transcriptome using *blastn* in BLAST at an e-value threshold of 1e^−10^. Contigs that did not result in a significant BLAST match were annotated using the non-redundant sequence database at an e-value threshold of 1e^−10^. Transcripts with putative gene products of bacterial and viral origin were removed whilst all remaining annotated contigs were concatenated to the 454 transcriptome, which we refer to as the ‘hybrid transcriptome’. To determine the completeness of the improved transcriptome, we mapped the assembled fur seal contigs against the most recent set of annotated dog transcripts [http://www.ncbi.nih.gov/genomes/Canis_familiaris/RNA/] using *blastn* in BLAST at an e-value threshold of 1e^−10^.

### SNP discovery

To mine SNPs from the hybrid transcriptome, we generated two *bam* files by mapping the raw Illumina paired-end reads to the hybrid transcriptome using both BWA and BOWTIE2 with the default parameters. Each mapping file was then parsed to GATK for SNP detection using the UnifiedGenotyper tool (-stand_call_conf 30, -stand_emit_conf 10). Each set of SNP calls was then hard-filtered using GATK’s VariantFiltration tool based on the following criteria: fisher strand bias <30, quality by depth >2, unfiltered read depth ≥10, read mapping quality ≥40. SNPs consequently flagged with anything other than ‘PASS’ were removed from the datasets. We also removed SNPs if read support for the minor allele was less than three.

In order to determine the extent of overlap between SNPs called by different methods, we revisited two sets of SNPs called from the 454 transcriptome using NEWBLER and SWAP454 respectively [33, 34]. A small number of SNPs within these datasets were duplicated or had an alternative allele frequency of one. These were therefore removed, leaving a total of 14,536 NEWBLER SNPs and 11,135 SWAP454 SNPs.

### SNP filtering and predicted assay success

We generated a global list of SNPs representing all of those called from (i) the 454 transcriptome using NEWBLER and SWAP454 and (ii) the hybrid transcriptome using BWA and BOWTIE2 in combination with GATK. We then implemented the steps outlined below to obtain subsets of SNPs suitable for designing Illumina Infinium iSelect HD and Affymetrix Axiom SNP assays respectively. Firstly, we used the BEDTOOLS command *getfasta* to extract the 121 bp SNP flanking sequences required for Illumina assays and the 71 bp flanking sequences required for Affymetrix assays. Loci with insufficient flanking sequence were discarded, as were a small number of SNPs that did not match the corresponding base in the genome sequence. The suitability of the resulting flanking sequences for each assay’s hybridization technology was then determined by generating Illumina Assay Design Tool (ADT) scores for the 121 bp SNP flanking sequences and Affymetrix p-convert scores for the 71 bp SNP flanking sequences. These were obtained from both Illumina and Affymetrix directly. SNPs assigned an ADT score of <0.8 were discarded from the Infinium dataset. For the Affymetrix dataset, SNPs with forward and/or reverse sequences designated ‘not recommended’ or ‘not possible’ were discarded.

For each SNP, we recorded the depth of coverage, minor allele frequency (MAF), ADT score (for Illumina assays) or p-convert score (for Affymetrix assays). We then mapped the corresponding Illumina and Affymetrix flanking sequences to the Antarctic fur seal reference genome [26] using *blastn* in BLAST with an e-value threshold of 1e^−12^. From this, we determined the alignment length of the top blast hit (a full and continuous mapping indicates that a SNP and its flanking sequences lie fully within an exon) and the total number of mappings (a proxy for sequence uniqueness).

Given the above information, we used two approaches to identify SNPs with high likelihoods of validation success for each SNP. First we simply filtered for SNPs whose flanking sequences match completely and uniquely to the genome, as these two characteristics have been shown to have a major affect on validation success [26]. Second, we used a predictive modeling approach based on the outcome of a pilot assay in which 144 putative fur seal SNPs were genotyped in 480 individuals [37]. Here, the known genotyping outcomes were used together with the genomic characteristics of the 144 SNP flanking sequences to construct a model of SNP validation success using *k*-fold cross validation. This approach splits the 144 observations into *k* = 5 non-overlapping subsets of approximately equal size, uses one subset as a validation sample and the remaining four subsets as training data in order to generate the best predictive model. This best model was then used to output the probability of each SNP successfully validating given values of the predictor variables using the *predict* function in the *bestglm* package in R [26]. A given SNP was predicted to validate successfully if its associated probability value was above 0.7.

## Additional file

10.1186/s13104-016-2209-x SNPs called by one, two, three or four methods, broken down by calling method, averaged across technology and filtering approach.
