# Supplementary material for: Transcriptomic SNP discovery for custom genotyping arrays: impacts of sequence data, SNP calling method and genotyping technology on the probability of validation success
Source: BMC Res Notes. 2016 Aug 26;9(1):418. doi: 10.1186/s13104-016-2209-x (PMC5000416; doi:10.1186/s13104-016-2209-x)
Supplement: Supplementary file 1 — 10.1186/s13104-016-2209-x SNPs called by one, two, three or four methods, broken down by calling method, averaged across technology and filtering approach. [file 13104_2016_2209_MOESM1_ESM.html]

Transcriptomic SNP discovery for a genotyping array


# Transcriptomic SNP discovery for a genotyping array

#### *Emily Humble*

This document provides the code for the main steps in our transcriptomic SNP discovery pipeline. It also includes code for generating the figures in the paper. Both the Rmarkdown file and the data required to run the code can be accessed by downloading this GitHub repository. Just click the link and then on *Download ZIP* at the right-hand side of the page. Part of this analysis relies on a large BLAST formatted genome database. You should download the full draft fur seal genome from Dryad and format it into a BLAST database. If you have any questions, please contact me: `emily.lhumble[at]gmail.com`

### Prepare the working environment

- If you have downloaded the `snp_filtering` project from github then you will see that:
- Raw data required are in `data/raw`
- R scripts sourced by this document are in `R/`
- You will also need to generate two new directories: `data/processed` and `data/blastdb`
- The BLAST formatted fur seal genome should be saved in `data/blastdb`. If you have downloaded the full draft genome, you can make it into a BLAST database using the following command: `makeblastdb -in final.assembly.ArcGaz001_AP3.fasta -dbtype nucl -out furseal`
- This pipeline invokes two system commands to run `blastn` and `bedtools`. The full path to working versions of these programs should be specfied in the script accordingly.
- The packages listed below are required. NOTE: The development version of `dplyr` is also required.

```
# uncomment the lines below to install the packages you need 

# source("http://bioconductor.org/biocLite.R")
# biocLite("VariantAnnotation")
# biocLite("ggbio")
# install.packages("gplots")
# if (packageVersion("devtools") < 1.6) {
#   install.packages("devtools")
# }
# library(devtools)
# devtools::install_github("hadley/lazyeval")
# devtools::install_github("hadley/dplyr")
# install.packages("tidyr")
# install.packages("MASS")
# install.packages("seqinr")
# install.packages("bestglm")
# install.packages("stringr")
# install.packages("DiagrammeR")
library(VariantAnnotation)
library(gplots)
library(dplyr)
library(tidyr)
library(seqinr)
library(bestglm)
library(stringr)
library(DiagrammeR)
library(ggplot2)
library(knitr)
library(magrittr)
library(pander)
library(ggbio)
```

---

### Load SNP data

- Import .vcf output files from **GATK** SNP calling that was carried out on **BOWTIE2** and **BWA** mapping files
- Remove SNPs where either the REF or ALT allele have read counts of < 3
- Apply function `manipvcf` to extract data from .vcf file and generate working dataframes `bowtie.snps` and `bwa.snps`
- See paper for SNP calling parameters

```
source("R/manip_vcf.R")
bowtie.snps <- readVcf("data/raw/highQualBowtieSNPs.vcf", "") %>%
  manipvcf() %>%
  filter(SNP_Count_RefAllele >=3 & SNP_Count_AltAllele >= 3) %>%
        mutate (Caller = "Bowtie2")

bwa.snps <- readVcf("data/raw/highQualBWASNPs.vcf", "") %>%
  manipvcf() %>%
  filter(SNP_Count_RefAllele >=3 & SNP_Count_AltAllele >= 3) %>%
        mutate (Caller = "BWA")

names(bowtie.snps)
```

- Import datasets from SNP calling carried out using **NEWBLER** and **SWAP454**
- Generate a Venn diagram to compare overlap between all four SNP discovery methods
- Combine datasets to generate a global list of SNPs: `universe`
- Determine how many methods a SNP was discovered by: `universe$share`

```
newbler.snps <- read.csv("data/raw/NewblerSNPs.csv")
swap454.snps <- read.csv("data/raw/swap454SNPs.csv")
```

```
universe <- bowtie.snps %>%
  full_join(bwa.snps, by = c("Contig_Name","SNP_Position","SNP_RefAllele",
                             "SNP_AltAllele"), all = T) %>%
  full_join(newbler.snps, by = c("Contig_Name", "SNP_Position", "SNP_RefAllele", 
                                 "SNP_AltAllele"), all = T) %>%
  full_join(swap454.snps, by = c("Contig_Name", "SNP_Position", "SNP_RefAllele", 
                                 "SNP_AltAllele"), all = T) %>%
        .[!duplicated(.[1:2]), ] %>%
        .[1:4] # devel v. of dplyr crashes my session

# get shared elements
universeID <- as.character(vennIDs(universe))
universe$share <- sapply(universeID, function(string) 
        sum(string==bowtieID, string==bwaID, string==newblerID, string==swap454ID)) # takes a while

length(universe[,1])
#> [1] 34718
```

### Exploring SNP parameter space

- Identify all SNPs called from `original` 454 sequencing data and all SNPs called from `illumina` sequencing data
- Generate a Venn diagram to compare overlap between both sequencing methods
- Identify those SNPs called from both sequencing methods: `intersect`

```
original <- newbler.snps %>%
        full_join(swap454.snps, by = c("Contig_Name", "SNP_Position"))
length(original[,1])
#> [1] 20426

illumina <- bowtie.snps %>%
        full_join(bwa.snps, by = c("Contig_Name", "SNP_Position")) 
length(illumina[,1])
#> [1] 18971

intersect <- newbler.snps %>%
        full_join(swap454.snps, by = c("Contig_Name", "SNP_Position")) %>%
        inner_join(illumina, by = c("Contig_Name", "SNP_Position"))
length(intersect[,1])
#> [1] 4679
```

- For SNPs called from the `original` transcriptome 18971, calculate mean *MAF* and *depth*
- For SNPs called from the `illumina` transcriptome 20426, calculate mean *MAF* and *depth*
- Repeat these steps for the SNPs called by both methods: `intersect` (4679)

```
depth_maf_454 <- matrix(ncol = 2, nrow = length(original[,1]))
depth_maf_454[,1] <- rowMeans(original[c(5,14)], na.rm=TRUE)
depth_maf_454[,2] <- rowMeans(original[c(8,18)], na.rm=TRUE)

depth_maf_454 <- data.frame(depth_maf_454)
names(depth_maf_454) <- c("Depth", "MAF")

depth_maf_illumina <- matrix(ncol = 2, nrow = length(illumina[,1]))
depth_maf_illumina[,1] <- rowMeans(illumina[c(7,16)], na.rm=TRUE)
depth_maf_illumina[,2] <- rowMeans(illumina[c(10,19)], na.rm=TRUE)

depth_maf_illumina <- data.frame(depth_maf_illumina)
names(depth_maf_illumina) <- c("Depth", "MAF")
```

- Use **MAF** and **depth** data to make marginal plots using the `my.filled.contour` function. This applies a kernel density function to the data with which it uses to make the contour plot. Histograms are plotted using the raw **MAF** and **depth** data.
- Repeat these steps for the SNPs called from both illumina and 454 sequencing data (4679). Code hidden in output.

```
source("R/my.filled.contour.R")
my.pal <- colorRampPalette(c("#e0ecf4","#bfd3e6","#9ebcda","#8c96c6",
                                 "#8c6bb1","#88419d","#810f7c","#4d004b"))
# 454 SNPs
k.454 <- with(depth_maf_454, MASS:::kde2d(log10(Depth), MAF, n = 29))

h1 <- hist(log10(depth_maf_454$Depth), breaks = 25, plot = F)
h2 <- hist(depth_maf_454$MAF, breaks = 25, plot = F)
top <- max(c(h2$counts, h1$counts))
 
oldpar <- par()
layout(matrix(c(2,0,1,3),2,2,byrow=T),c(3,1), c(1,3), TRUE) # set layout for plot

# make contour plot
par(mar=c(3,3,1,1))
my.filled.contour(k.454, color.palette = my.pal)

# add histograms
par(mar=c(0,2,1,0)) # (lower height, left width , upper height , rigth width)
barplot(h1$counts, axes=F, ylim=c(0, top), space=0, col = "#bfd3e6")
par(mar=c(1.75,0,0,1)) #c(1,0,0.5,1))
barplot(h2$counts, axes=F, xlim=c(0, top), space=0, col='#bfd3e6', horiz=T)

# Illumina SNPs
k.illumina <- with(depth_maf_illumina, MASS:::kde2d(log10(Depth), MAF, n = 29))

h1 <- hist(log10(depth_maf_illumina$Depth), breaks = 25, plot = F)
h2 <- hist(depth_maf_illumina$MAF, breaks = 25, plot = F)
top <- max(c(h2$counts, h1$counts))

oldpar <- par()
layout(matrix(c(2,0,1,3),2,2,byrow=T),c(3,1), c(1,3), TRUE) # set layout for plot

# make contour plot
par(mar=c(3,3,1,1))
my.filled.contour(k.illumina, color.palette = my.pal)

# add histograms
par(mar=c(0,2,1,0)) # (lower height, left width , upper height , rigth width)
barplot(h1$counts, axes=F, ylim=c(0, top), space=0, col = "#bfd3e6")
par(mar=c(1.75,0,0,1))
barplot(h2$counts, axes=F, xlim=c(0, top), space=0, col='#bfd3e6', horiz=T)
```

Depth of coverage is on the **x** axis and minor allele frequency is on the **y** axis. The upper panels correspond to the total number of SNPs called from the 454 (top left) and illumina data (top right). The lower panels correpsond to the 4679 SNPs that were called from both the 454 and illumina datasets. The left plots shows the 454 parameter space and the right plots shows the corresponding illumina parameter space.

---

### Filtering for a SNP array

Having explored the SNPs called across methods, we now take the full set of SNPs, `universe`, and filter for suitability for both an **Illumina Infinium HD II** and an **Affymetrix Axiom** genotyping array.

### 1. Extract flanking sequences

- Using the functions `get_illuminaflanks` and `get_axiomflanks`, identify the start and end positions of the 61 bp `infinium` and 35 bp `axiom` flanking sequences
- Remove SNPs in which flanking sequence extraction from the transcriptome is not be possible i.e. SNP lies too close to the start or end of the contig

```
# load transcript length data
lengths <- read.table("data/raw/transcriptlenghts.txt", header = T)
source("R/getsnpflanking.R")

infinium <- get_illuminaflanks(universe, lengths) %>%
        filter(SNPenclose == 61 & SNP_Position < Length & two - SNP_Position == 60)
length(infinium[,1])
#> [1] 31192

axiom <- get_axiomflanks(universe, lengths) %>%
        filter(SNPenclose == 36 & SNP_Position < Length & two - SNP_Position == 35)
length(axiom[,1])
#> [1] 32727
```

- Generate .bed files using base positions generated above
- Extract the flanking sequences from the fur seal transcriptome using `bedtools` and import them into R

```
write.table(infinium[c(1,3,4)], "data/processed/infiniumflanks.bed", 
            quote = F, col.names = F, row.names = F, sep = "\t")

# run bed tools
bedCmd <- paste("~/programs/bedtools getfasta -fi data/raw/joined_transcriptome.fasta -bed data/processed/infiniumflanks.bed -fo data/processed/infiniumflanks.fasta")
system(bedCmd)

infinium <- read.fasta("data/processed/infiniumflanks.fasta", as.string = T, forceDNAtolower = F) %>%
        lapply(function(x) x[[1]]) %>%
        as.character(unlist(.)) %>%
        mutate(infinium, seq = .) %>%
        .[c(1:2,6:7)] %>%
        left_join(universe)
length(infinium[,1])
#> [1] 31192
```

- Do the same for the `axiom` data

```
write.table(axiom[c(1,3,4)], "data/processed/axiomflanks.bed", 
            quote = F, col.names = F, row.names = F, sep = "\t")

# run bed tools

bedCmd <- paste("~/programs/bedtools getfasta -fi data/raw/joined_transcriptome.fasta -bed data/processed/axiomflanks.bed -fo data/processed/axiomflanks.fasta")
system(bedCmd)

axiom <- read.fasta("data/processed/axiomflanks.fasta", as.string = T, forceDNAtolower = F) %>%
        lapply(function(x) x[[1]]) %>%
        as.character(unlist(.)) %>%
        mutate(axiom, seq = .) %>%
        .[c(1:2,6:7)] %>%
        left_join(universe)
length(axiom[,1])
#> [1] 32727
```

### 2. Get company design scores

In order to generate ADT scores and p-convert scores, SNP flanking sequences must be submitted to Illumina and Affymetrix.

- Use functions `getPconvertfile` and `getADT` to generate the necessary files for submission to each manufacturer

```
source("R/arraydesign_seq.R")
getADTfile(infinium)
getPconvertfile(axiom)
```

The files returned by each manufacturer are stored in `output/design_scores`

- Import files and add useful information to each SNP dataset
- Retain `infinium` SNPs with an ADT score above 0.8
- Retain `axiom` SNPs with at least “recommended” or “neutral” in both strands

```
infinium <- read.csv("output/design_scores/WGGTPrelimScoreResults.csv", skip = 20, header =T) %>%
        dplyr::select(Locus_Name, Final_Score) %>%
        mutate(Locus_Name = gsub("_v", "v", Locus_Name)) %>%
        separate(Locus_Name, c("Contig_Name", "SNP_Position"), sep = "\\_") %>%
        mutate(Contig_Name = gsub("v", "_v", Contig_Name)) %>%
        mutate(Contig_Name = as.factor(Contig_Name), SNP_Position = as.integer(SNP_Position)) %>%
        left_join(infinium, by = c("Contig_Name", "SNP_Position")) %>%
        filter(Final_Score >= 0.8) %>%
        dplyr::select(Contig_Name, SNP_Position, seq, SNP_RefAllele, SNP_AltAllele, Final_Score, share)

length(infinium[,1])
#> [1] 26110

axiom <- read.table("output/design_scores/Axiom_FurSeal_recommendation.txt", header =T) %>%
        mutate(forwardPconvert = as.character(forwardPconvert), reversePconvert = as.character(reversePconvert)) %>%
        mutate(forwardPconvert = as.numeric(forwardPconvert), reversePconvert = as.numeric(reversePconvert)) %>%
        dplyr::select(SNPId, forwardPconvert, forwardRecommendation, reversePconvert, reverseRecommendation) %>%
        separate(SNPId, c("Contig_Name", "SNP_Position"), sep = "\\_") %>%
        mutate(Contig_Name = gsub("v", "_v", Contig_Name)) %>%
        mutate(Contig_Name = as.factor(Contig_Name), SNP_Position = as.integer(SNP_Position)) %>%
        left_join(axiom, by = c("Contig_Name", "SNP_Position")) %>%
        filter(forwardRecommendation %in% c("recommended", "neutral") | reverseRecommendation %in% c("recommended", "neutral")) %>%
        mutate(pconvert = (forwardPconvert + reversePconvert) / 2) %>%
        dplyr::select(Contig_Name, SNP_Position, seq, SNP_RefAllele, SNP_AltAllele, pconvert, share)

length(axiom[,1])
#> [1] 24778
```

### 3. Determine SNP flanking sequence genomic context

Here we use the draft fur seal genome to determine genomic characteristics of the SNP flanking sequences. We can then use this information to select the SNPs we think will perform well on a SNP array based on their genomic context.

- Use the function `get.fasta` to generate flanking sequence fasta files from each of the `infinium` and `axiom` datasets to blast against the genome

```
get.fasta <- function(x){
  names <- do.call(paste, c(x[c(1,2)], sep = "_"))
  names <- data.frame(paste(">", names, sep = ""))
  fasta <- cbind(names, x$seq)
  fasta <- as.vector(t(fasta))
  fasta <- as.data.frame(fasta)
  write.table(fasta, paste("data/processed/", substitute(x), ".fasta", sep = ""), quote = F, col.names = F, row.names = F)
}

get.fasta(infinium)
get.fasta(axiom)
```

- BLAST the flanking sequences against the genome using `blastn`

You need to download the full genome from here, `blastdb` format it, and save it in `data/blastdb/`

```
infilenames <- list.files(path = "data/processed", pattern = "*.fasta")
outnames <- paste(unlist(sapply(infilenames, strsplit, split = "*.fasta")),
                  "SNPs2genome", sep = "")

# function to create the commands
cmdCreate <- function(infile, outfile){
  paste("~/programs/blastn -db data/blastdb/furseal -outfmt 6 -num_threads 32 -evalue 1e-12 -query data/processed/",infile, " -out data/processed/",
        outfile, sep = "")
}

# create the commands
cmds <- mapply(FUN = cmdCreate, infile = infilenames, outfile = outnames)

# run the blasts. this will obviously take a while when using the full genome

# sapply(cmds, system)
```

### 4. Predict SNP validation outcomes

- Create dataframes containing SNP metadata and genome BLAST results for both `infinium` and `axiom` SNPs. We will use these to evaluate SNP genomic context and predict SNP validation outcomes
- The development version of `dplyr` is required for this code to produce the correct output. If for some reason you cannot get it to work, you can import the dataframes I made earlier instead.

```
# These pipes require development version of dplyr

infinium <- infinium %>%
        mutate(Contig_Name = as.character(Contig_Name)) %>%
        dplyr::left_join(bowtie.snps[, c("Contig_Name", "SNP_Position", "AlignDepth", "MAF")], 
                         by = c("Contig_Name", "SNP_Position")) %>%
        dplyr::left_join(bwa.snps[, c("Contig_Name", "SNP_Position", "AlignDepth", "MAF")], 
                         by = c("Contig_Name", "SNP_Position")) %>%
        dplyr::left_join(newbler.snps[, c("Contig_Name", "SNP_Position", "AlignDepth", "MAF")], 
                         by = c("Contig_Name", "SNP_Position")) %>%
        dplyr::left_join(swap454.snps[, c("Contig_Name", "SNP_Position", "AlignDepth", "MAF")], 
                         by = c("Contig_Name", "SNP_Position"))

axiom <- axiom %>%
        mutate(Contig_Name = as.character(Contig_Name)) %>%
        dplyr::left_join(bowtie.snps[, c("Contig_Name", "SNP_Position", "AlignDepth", "MAF")], 
                         by = c("Contig_Name", "SNP_Position")) %>%
        dplyr::left_join(bwa.snps[, c("Contig_Name", "SNP_Position", "AlignDepth", "MAF")], 
                         by = c("Contig_Name", "SNP_Position")) %>%
        dplyr::left_join(newbler.snps[, c("Contig_Name", "SNP_Position", "AlignDepth", "MAF")], 
                         by = c("Contig_Name", "SNP_Position")) %>%
        dplyr::left_join(swap454.snps[, c("Contig_Name", "SNP_Position", "AlignDepth", "MAF")], 
                         by = c("Contig_Name", "SNP_Position"))
       
# Dataframes I made earlier in case you can't get devel version to work. Sometimes it crashes my Rstudio session
# infinium <- read.csv("data/some_i_made_earlier/infinum_df_develdplyr.csv", header = T)
# axiom <- read.csv("data/some_i_made_earlier/axiom_df_develdplyr.csv", header = T)

source("R/make_dfs.R")
source("R/blasthitspersnp.R")
infinium_df <- make_model_df(infinium)
axiom_df <- make_model_df(axiom)
```

- Read in BLAST output and merge with `axiom` and `infinium` dataframes so that we can assess SNP genomic context

```
# infinium_df <- read.table("data/processed/infiniumSNPs2genome") %>%
#         blasthitspersnp(., infinium_df)
 
# axiom_df <- read.table("data/processed/axiomSNPs2genome") %>%
#         blasthitspersnp(., axiom_df)

# Files I merged earlier (using blast against the full genome)
infinium_df <- read.csv("data/some_i_made_earlier/infinium_dfwithblast.csv", header = T)
axiom_df <- read.csv("data/some_i_made_earlier/axiom_dfwithblast.csv", header = T)
```

First, we use a predictive method based on the results of a pilot study to predict the outcome of our SNPs on a SNP array given their genomic context.

- Load the pilot data. This is a dataframe containing genome blast results, company design scores, depth of coverage, minor allele frequency (MAF), number of genome mappings and validation outcome (y) for 142 SNPs
- Generate best predictive model based on these data using 5-fold cross validation

```
# Load pilot dataframe
pilot <- read.csv("data/raw/pilot/furseal_pilot.csv", header = T)
pilot <- na.omit(pilot)
pilot_infinium <- pilot[c(2,6,7,10,11,12,13)]
pilot_axiom <- pilot[c(2,6,7,10,11,12,13)]
head(pilot_infinium)
#>   AlignmentLength BitScore pconvert depth       MAF Count y
#> 1             113      209    0.659    41 0.1951220     1 1
#> 2             121      224    0.667    40 0.4250000     1 1
#> 3             121      224    0.645    32 0.4687500     2 0
#> 4             119      220    0.669    20 0.4500000     4 0
#> 5             121      224    0.658    30 0.3666667     1 1
#> 6             121      224    0.650    39 0.2820513     1 1

# Generate best predictive model
infinium_model <- bestglm(pilot_infinium, family = binomial, IC = "CV", CVArgs = list(Method = "HTF", K = 5, REP = 100))
#> Morgan-Tatar search since family is non-gaussian.
axiom_model <- bestglm(pilot_axiom, family = binomial, IC = "CV", CVArgs = list(Method = "HTF", K = 5, REP = 100))
#> Morgan-Tatar search since family is non-gaussian.
```

- Use the best model to predict the validation outcomes of the `infinium` and `axiom` SNPs
- Identify SNPs that have at least a 70% probability of validation success

```
# Prediction
model <- infinium_model
df <- infinium_df

pred_mod <- function(model, df){
        glm.probs <- predict(model$BestModel, df, type = "response")
        thresh <- 0.7
        glm.pred <- cut(glm.probs, breaks=c(-Inf, thresh, Inf), labels = c("Low", "High"))
        predictions <- as.data.frame(glm.pred)
        fail <- subset(predictions, predictions$glm.pred == "Low")
        pass <- subset(predictions, predictions$glm.pred == "High")
        df$outcome <- glm.pred
        success <- subset(df, df$outcome == "High")
        rate <- (length(predictions$glm.pred)-length(fail$glm.pred))/length(predictions$glm.pred)
        cat("Filtering based on predictive modeling")
        cat("\nNumber of SNPs predicted to succesfully validate:", length(pass[,1]))
        cat("\nNumber of SNPs predicted to fail:", length(fail[,1]))
        cat("\nPredicted validation success rate:", rate)
        success <- success
}

# RESULTS
#------------------------------------------------
# Infinium SNPs
infinium_model_pass <- pred_mod(infinium_model, infinium_df)
#> Filtering based on predictive modeling
#> Number of SNPs predicted to succesfully validate: 19773
#> Number of SNPs predicted to fail: 4474
#> Predicted validation success rate: 0.8154823

# Axiom SNPs
axiom_model_pass <- pred_mod(axiom_model, axiom_df)
#> Filtering based on predictive modeling
#> Number of SNPs predicted to succesfully validate: 21141
#> Number of SNPs predicted to fail: 1227
#> Predicted validation success rate: 0.9451448
```

### Crude filtering

When it is not possible to use a pre-validated set of SNPs for prediction purposes, you can apply a more crude filtering approach based on two of the most important genomic characteristics. One can then assume that these SNPs will have a high probability of validation success.

- Filter datasets to include only SNPs that map uniquely and completely to the genome

```
crude_filt <- function(x){
        x["seq"] <- as.character(x["seq"])
        x["probelength"] <- str_length(x["seq"])
        good <- TRUE
        if (x["AlignmentLength"] != x["probelength"]) good <- FALSE
        good
}

crude_results <- function(x){
        success <- filter(x, goodness == TRUE & Count == 1)
        cat("Crude Filtering")
        cat("\nNumber of SNPs with good genomic characteristics:", length(success[,1]))
        cat("\nProportion of total:", length(success[,1]) / length(x[,1]))
}

# RESULTS
#------------------------------------------------
# Infinium SNPs
snp_goodness <- apply(infinium_df, 1, crude_filt)
infinium_df$goodness <- snp_goodness
crude_results(infinium_df)
#> Crude Filtering
#> Number of SNPs with good genomic characteristics: 11057
#> Proportion of total: 0.4560152

# Axiom SNPs
snp_goodness <- apply(axiom_df, 1, crude_filt)
axiom_df$goodness <- snp_goodness
crude_results(axiom_df)
#> Crude Filtering
#> Number of SNPs with good genomic characteristics: 14901
#> Proportion of total: 0.6661749

# Extract the good SNPs
infinium_filter_pass <- subset(infinium_df, infinium_df$goodness == TRUE & Count == 1)
axiom_filter_pass <- subset(axiom_df, axiom_df$goodness == TRUE & Count == 1)
```

---

### Tables

The following code generates the summary data presented in Table 1 and Table 2 of the paper

```
source("R/parse_results.R")

# Table 2
# Proportion of those SNPs shared by one, two, three and four calling methods predicted to successfully validate on both an Illumina Infinium and an Affymetrix Axiom array using using predictive modeling and simple filtering approaches. 

share_outcomes <- get_overall_outcomes(infinium_df, axiom_df, 
                                       infinium_model_pass, infinium_filter_pass, 
                                       axiom_model_pass, axiom_filter_pass)

# Table 1
# Proportion of SNPs from each discovery method predicted to successfully validate on both an Illumina Infinium and an Affymetrix Axiom array using predictive modeling and simple filtering approaches.

initial_SNPs <- c(15109, 18353, 14538, 11155)

caller_outcomes <- get_caller_outcomes(infinium_model_pass, infinium_filter_pass, 
                                axiom_model_pass, axiom_filter_pass)

caller_share <- melt(caller_outcomes, id.vars=c("share", "total", "tech", "method", "success_rate"), 
                         measure.vars = c("bowtie", "bwa", "newbler", "swap454"))

caller_share <- caller_share %>%
        group_by(tech, method, variable) %>%
        mutate(value = as.numeric(as.character(value))) %>%
        summarise(total_success = sum(value)) %>%
        mutate(initial = initial_SNPs) %>%
        mutate(prop_successful = (total_success/initial) * 100)
```

Table 1. Proportion of SNPs from each discovery method predicted to successfully validate on both an Illumina Infinium and an Affymetrix Axiom array using predictive modeling and simple filtering approaches


| tech | method | variable | prop\_successful |
| --- | --- | --- | --- |
| ax | filt | bowtie | 61.69833 |
| ax | filt | bwa | 54.62322 |
| ax | filt | newbler | 35.46568 |
| ax | filt | swap454 | 45.88974 |
| ax | mod | bowtie | 83.34767 |
| ax | mod | bwa | 78.52667 |
| ax | mod | newbler | 48.92695 |
| ax | mod | swap454 | 61.77499 |
| inf | filt | bowtie | 45.60858 |
| inf | filt | bwa | 39.74827 |
| inf | filt | newbler | 27.28711 |
| inf | filt | swap454 | 34.60332 |
| inf | mod | bowtie | 75.69660 |
| inf | mod | bwa | 72.09720 |
| inf | mod | newbler | 46.83588 |
| inf | mod | swap454 | 56.97893 |

Table 2. Proportion of those SNPs shared by one, two, three and four calling methods predicted to successfully validate on both an Illumina Infinium and an Affymetrix Axiom array using using predictive modeling and simple filtering approaches


| share | tech | method | success\_rate |
| --- | --- | --- | --- |
| 1 | inf | mod | 66.75 |
| 2 | inf | mod | 92.92 |
| 3 | inf | mod | 89.34 |
| 4 | inf | mod | 89.87 |
| 1 | inf | filt | 41.46 |
| 2 | inf | filt | 54.69 |
| 3 | inf | filt | 48.62 |
| 4 | inf | filt | 50.7 |
| 1 | ax | mod | 67.94 |
| 2 | ax | mod | 101.2 |
| 3 | ax | mod | 100.5 |
| 4 | ax | mod | 99.41 |
| 1 | ax | filt | 56.98 |
| 2 | ax | filt | 72.59 |
| 3 | ax | filt | 68.08 |
| 4 | ax | filt | 68.23 |

---

### Overview

This figure shows the number of SNPs remaining after each of the main filtering steps. Blue circles represent `infinium` SNPs and purple circles represent `axiom` SNPs.

### Additional Figures

The following code generates the Circos Plot

```
# Prepare illumina coverage data-----------------------------------------------
# Read in average transcript coverage for bwa and bowtie mapping
# Get overall average mapping coverage across both methods
# Get old contig ID name
# Mask coverage values less than 10 (SNPs were called at this cutoff)

full_illum_cov <- read.csv("data/raw/mapping/bwa_coverage.csv", header = T) %>%
        full_join((read.csv("data/raw/mapping/bowtie_coverage.csv")), by = "comps") %>%
        mutate(coverage.x = ifelse(is.na(coverage.x), 0, coverage.x),
               coverage.y = ifelse(is.na(coverage.y), 0, coverage.y)) %>%
        mutate(Average.Depth.Illumina = (coverage.x + coverage.y) / 2) %>%
        select(comps, Average.Depth.Illumina) %>%
        set_colnames(c("New_Contig_ID", "Average.Depth.Illumina")) %>%
        left_join((read.csv("other/genemap.csv")), by = "New_Contig_ID") %>%
        mutate(Average.Depth.Illumina = replace(Average.Depth.Illumina, Average.Depth.Illumina < 10, NA))

# Prepare dataframe for 454 transcriptome--------------------------------------
# Join 454 coverage with illumina read coverage data
# Arrange in order of decreasing illumina coverage
# Select 454 transcripts
# Get values for IRanges

orig_cov <- read.csv("data/raw/mapping/assembly_1st_round_newbler.ace.txt_stats.csv", header = T) %>%
        rbind(read.csv("data/raw/mapping/assembly_2nd_round_cap3.ace.txt_stats.csv", header = T)) %>%
        full_join(full_illum_cov, by = c("Name" = "Orig_Contig_ID")) %>%
        right_join((read.table("data/raw/transcriptlenghts.txt", header = T)), by = c("New_Contig_ID" = "Contig_Name")) %>%
        .[c(1:23096),c(3,6,7,8)] %>%
        arrange(desc(Average.Depth.Illumina)) %>%
        mutate(end_range = cumsum(Length)) %>%
        mutate(start_range = end_range + 1) %>%
        mutate(start_range=lag(start_range)) %>%
        mutate(start_range = replace(start_range, is.na(start_range), 1))
        
orig_cov[is.na(orig_cov)] <- 0  # 23096

# percent of original transcriptome with insufficent coverage for illumina SNP calling
sum(orig_cov$Average.Depth.Illumina==0) / nrow(orig_cov) * 100
#> [1] 38.44822

# Prepare dataframe for illumina transcriptome---------------------------------
# Join 454 coverage with illumina read coverage data
# Arrange in order of decreasing illumina coverage
# Select illumina transcripts
# Get values for IRanges

illum_cov <- read.csv("data/raw/mapping/assembly_1st_round_newbler.ace.txt_stats.csv", header = T) %>%
        rbind(read.csv("data/raw/mapping/assembly_2nd_round_cap3.ace.txt_stats.csv", header = T)) %>%
        full_join(full_illum_cov, by = c("Name" = "Orig_Contig_ID")) %>%
        right_join((read.table("data/raw/transcriptlenghts.txt", header = T)), by = c("New_Contig_ID" = "Contig_Name")) %>%
        .[c(23097:28548),c(3,6,7,8)] %>%
        arrange(desc(Average.Depth.Illumina)) %>%
        mutate(end_range = cumsum(Length)) %>%
        mutate(start_range = end_range + 1) %>%
        mutate(start_range=lag(start_range)) %>%
        mutate(start_range = replace(start_range, is.na(start_range), 1))

illum_cov[is.na(illum_cov)] <- 0 # 5452

# Join two dataframes together and log coverage values-------------------------

cov <- rbind(orig_cov, illum_cov) %>%
        mutate(Log.Average.Depth.Illumina=log10(Average.Depth.Illumina)) %>%
        mutate(Log.Average.Depth=log10(Average.Depth)) %>%
        mutate(Log.Average.Depth = gsub("-Inf", 0, Log.Average.Depth)) %>%
        mutate(Log.Average.Depth.Illumina = gsub("-Inf", 0, Log.Average.Depth.Illumina)) %>%
        mutate(Log.Average.Depth.Illumina = as.numeric(Log.Average.Depth.Illumina)) %>%
        mutate(Log.Average.Depth = as.numeric(Log.Average.Depth)) 

# Generate the circos plot

gr <- GRanges(seqnames = c('chr1','chr2'),
              IRanges(start = 1,
                      end = c(22425577, 4682077)),
                      Transcriptome = sample(c('454','Illumina'), size = 2))


data <- GRanges(seqnames = c(rep('chr1', 23096), rep('chr2', 5452)),
                IRanges(start = cov$start_range, 
                        end = cov$end_range), # width?
                d= cov$Log.Average.Depth,
                d2 = cov$Log.Average.Depth.Illumina,
                e = cov$Average.Depth,
                e2 = cov$Average.Depth.Illumina)


mycolours <- c("#9ebcda", "#8c6bb1")
#pdf("output/figs/Figure_1.pdf", width = 11, height = 11)
print(ggbio() + circle(gr,  geom = 'rect', aes(fill = Transcriptome), space.skip = 0.01) + scale_fill_manual(values=mycolours) + 
               circle(data , geom  = 'bar', aes(y = d), colour = "#8c6bb1" , fill = "#8c6bb1", trackWidth = 10) +
               circle(data , geom  = 'bar', aes(y = d2), colour = "#9ebcda" , fill = "#9ebcda", trackWidth = 13))
#dev.off()
```

---

R version and platform.

```
sessionInfo()
#> R version 3.2.3 (2015-12-10)
#> Platform: x86_64-apple-darwin13.4.0 (64-bit)
#> Running under: OS X 10.10.5 (Yosemite)
#> 
#> locale:
#> [1] en_GB.UTF-8/en_GB.UTF-8/en_GB.UTF-8/C/en_GB.UTF-8/en_GB.UTF-8
#> 
#> attached base packages:
#> [1] stats4    parallel  stats     graphics  grDevices utils     datasets 
#> [8] methods   base     
#> 
#> other attached packages:
#>  [1] pander_0.6.0              ggbio_1.16.1             
#>  [3] BiocInstaller_1.18.5      magrittr_1.5             
#>  [5] knitr_1.13                ggplot2_2.1.0            
#>  [7] stringr_1.0.0             bestglm_0.34             
#>  [9] leaps_2.9                 reshape_0.8.5            
#> [11] seqinr_3.1-5              ade4_1.7-4               
#> [13] DiagrammeR_0.8.2          reshape2_1.4.1           
#> [15] tidyr_0.5.0               dplyr_0.4.3              
#> [17] gplots_3.0.1              VariantAnnotation_1.14.13
#> [19] Rsamtools_1.20.5          Biostrings_2.36.4        
#> [21] XVector_0.8.0             GenomicRanges_1.20.8     
#> [23] GenomeInfoDb_1.4.3        IRanges_2.2.9            
#> [25] S4Vectors_0.6.6           BiocGenerics_0.14.0      
#> 
#> loaded via a namespace (and not attached):
#>  [1] bitops_1.0-6            RColorBrewer_1.1-2     
#>  [3] tools_3.2.3             R6_2.1.2               
#>  [5] rpart_4.1-10            KernSmooth_2.23-15     
#>  [7] Hmisc_3.17-4            DBI_0.4-1              
#>  [9] lazyeval_0.2.0          colorspace_1.2-6       
#> [11] nnet_7.3-12             gridExtra_2.2.1        
#> [13] GGally_1.0.1            chron_2.3-47           
#> [15] graph_1.46.0            Biobase_2.28.0         
#> [17] formatR_1.4             rtracklayer_1.28.10    
#> [19] caTools_1.17.1          scales_0.4.0           
#> [21] RBGL_1.44.0             digest_0.6.9           
#> [23] foreign_0.8-66          rmarkdown_0.9.6        
#> [25] dichromat_2.0-0         htmltools_0.3.5        
#> [27] BSgenome_1.36.3         htmlwidgets_0.6        
#> [29] rstudioapi_0.5          RSQLite_1.0.0          
#> [31] visNetwork_1.0.0        jsonlite_0.9.21        
#> [33] BiocParallel_1.2.22     gtools_3.5.0           
#> [35] acepack_1.3-3.3         RCurl_1.95-4.8         
#> [37] Formula_1.2-1           futile.logger_1.4.1    
#> [39] Matrix_1.2-6            Rcpp_0.12.5            
#> [41] munsell_0.4.3           stringi_1.1.1          
#> [43] yaml_2.1.13             zlibbioc_1.14.0        
#> [45] plyr_1.8.4              grid_3.2.3             
#> [47] gdata_2.17.0            lattice_0.20-33        
#> [49] splines_3.2.3           GenomicFeatures_1.20.6 
#> [51] codetools_0.2-14        biomaRt_2.24.1         
#> [53] futile.options_1.0.0    XML_3.98-1.4           
#> [55] evaluate_0.9            biovizBase_1.16.0      
#> [57] latticeExtra_0.6-28     lambda.r_1.1.7         
#> [59] data.table_1.9.6        gtable_0.2.0           
#> [61] assertthat_0.1          survival_2.39-4        
#> [63] tibble_1.0              OrganismDbi_1.10.0     
#> [65] GenomicAlignments_1.4.2 AnnotationDbi_1.30.1   
#> [67] cluster_2.0.4
```
